# Evolution of scaling behaviors embedded in sentence series from *A Story of the Stone*

**DOI:** 10.1371/journal.pone.0171776

**Published:** 2017-02-14

**Authors:** Yue Yang, Changgui Gu, Qin Xiao, Huijie Yang

**Affiliations:** 1 Business School, University of Shanghai for Science and Technology, Shanghai 200093, China; 2 College of Sciences, Shanghai Institute of Technology, Shanghai 201418, China; Tianjin University, CHINA

## Abstract

The novel entitled *A Story of the Stone* provides us precise details of life and social structure of the 18th century China. Its writing lasted a long duration of about 10 years, in which the author’s habit may change significantly. It had been published anonymously up to the beginning of the 20th century, which left a mystery of the author’s attribution. In the present work we focus our attention on scaling behavior embedded in the sentence series from this novel, hope to find how the ideas are organized from single sentences to the whole text. Especially we are interested in the evolution of scale invariance to monitor the changes of the author’s language habit and to find some clues on the author’s attribution. The sentence series are separated into a total of 69 non-overlapping segments with a length of 500 sentences each. The correlation dependent balanced estimation of diffusion entropy (cBEDE) is employed to evaluate the scaling behaviors embedded in the short segments. It is found that the total, the part attributed currently to Xueqin Cao (X-part), and the other part attributed to E Gao (E-part), display scale invariance in a large scale up to 10^3^ sentences, while their scaling exponents are almost identical. All the segments behave scale invariant in considerable wide scales, most of which reach one third of the length. In the curve of scaling exponent versus segment number, the X-part has rich patterns with averagely larger values, while the E-part has a *U*-shape with a significant low bottom. This finding is a new clue to support the attribution of the E-part to E Gao.

## Introduction

Structural patterns in language can give clues on how our brains process information, how our society is organized and shared, and how the world is structured [1–4]. They provide us also quantitative measures to distinguish habit styles of different authors and to monitor the development of linguistic abilities. A quantitative investigation on human language can trace back to the Zipf’s law [5], namely, the occurring frequency of a most frequent word in a text obeys generally a power-law according to its rank, if we rank the words in a descending order of their occurring frequencies. This work has stimulated great efforts on discovering structural patterns embedded in texts. For instance, all the distances between successive occurrences of a word form a distance series. The distance distributes with a stretched exponential function [6, 7], and the distance series is long-range correlated [8–11]. Herein a distance refers to the number of words (characters); If we take each distinguished word as a node and link every pair of nodes that occur successively, this procedure will generate a typical small-world network [12–14](a small average distance between the words and a high probability of a node’s neighbors being linked also).

The focus of the mentioned findings is on the words in a text. In a very recent paper [15], the statistical properties of punctuation marks were investigated in detail. It is found that they obey also the Zipf’s law, and adding them as words to Zipfian analysis restores the power-law Zipfian behavior from a shifted power-law. Besides the grammatical role, a punctuation carries also some semantic load similar to that of the articles, conjunctions, pronouns and prepositions. Hence, it is necessary to incorporate punctuation marks in a language analysis, which opens more space for new observations.

Here we pay specially our attentions on the punctuation marks indicating end of a sentence. A sentence is the natural unit of a language. From several sentences as a context, one can restrict a word to an exact meaning. Sentences are organized logically into meaning groups, paragraphs, chapters and the whole text to display our ideas with a concise structure. The sequential order of sentence is a cooperative result of logic, fluency, rhythm, harmony, intonation and author’s style, etc.

One of the important properties of a sentence series is its scaling behavior. Let us consider a sentence series denoted with,

$$\xi =\{\xi_{1},\xi_{2},\cdots ,\xi_{N}\},$$ (1)

from which one can extract all the possible segments with a predefined length *s*,

$$\Xi_{n}^{s}=\left\{\xi_{n},\xi_{n+1},\cdots ,\xi_{n+s-1}\right\},n=1,2,\cdots ,N-s+1.$$ (2)

Here *ξ*_*n*_ is the number of words (characters) contained in the *n*th sentence. Now we regard each segment as a trajectory in a duration of *s*, starting from the original point. The sentence series is mapped to an ensemble of *N* − *s* + 1 realizations of a stochastic process, whose displacements read,

$$x_{n}\left(s\right)\equiv \sum_{j=1}^{s}\Xi_{n}^{s}\left(j\right)=\sum_{j=n}^{n+s-1}\xi_{n},n=1,2,\cdots ,N-s+1.$$ (3)

The stochastic process can be described with the probability distribution function (PDF) of the displacements, *p*(*x*, *s*). The sentence series is called to be scale invariant if the PDF satisfies a special form [16],

$$p\left(x,s\right)∼\frac{1}{s^{\delta }}F\left(\frac{x}{s^{\delta }}\right),$$ (4)

namely, its shape keeps unchanged under a re-scale procedure of displacements, $x\to \frac{x}{s^{\delta }}$. A scale invariance implies that our ideas at different levels are structured with identical rules.

Drozdz et al. [17] found that more than one hundred classical novels all over the world shared a universal characteristic of almost a perfect long-range correlation. For all the sentence series constructed from the novels, the autocorrelation coefficient decays according to a power-law. Yang et al. [18] found also the scale invariant behavior in the sentence series from *A story of the Stone*, the scaling exponent of which turns out to be similar with that of the *New Testament* in the *Holy Bible* [17].

A language evolves with time and environmental conditions, the characteristics of which may be stored in the classics completed in long durations. For an example, the novel entitled *A Story of the Stone* is one of the the top four masterpieces in Chinese novels [19], and famous for its great value for researchers from diverse research fields. Its writing lasted about 10 years, during which the social environment and personal circumstances for the author(s) had changed significantly, which may lead to changes of language habits. In the present paper, we will separate the sentence series from *A Story of the Stone* into many segments as being the representatives of the habits in the corresponding durations, and evaluate the scaling behavior embedded in every segment, from which we hope to find the evolutionary behaviors of the author’s language habit.

What is more, the novel was initially published anonymously up to the beginning of the 20th century, which left some arguments on the author’s attribution [19]. Current opinion is that the part from the 1st to the 80th chapter was written by Xueqin Cao, while the other part from the 81th to the 120th chapter was written by E Gao, though there are still some debates up to now. By monitoring the evolutionary behavior of the habit, we hope also to find some clues on the author’s attribution.

In literature, there exist some standard tools to evaluate scaling behaviors of time series, such as the wavelet transformation modulus maxima (WTMM) [20–22] and the de-trended fluctuation analysis (DFA) [23–30]. But when we try to find scaling behaviors embedded in the segments of sentence series, one will meet several essential problems [31–33]. First, these variance based methods are dependent with dynamical processes. For a fractional Brownian motion, they can find the scale invariant behavior and the estimated scaling exponent *H* equals to the real one, namely, *H* = *δ*. For a Levy walk, the power-law of the variance versus the scale *s* can be found but it’s scaling exponent *H* does not equal to *δ*. The relation between them reads, $\delta =\frac{1}{3-2H}$ [33]. For a Levy flight motion, one can not detect qualitatively out the scale invariance. We have almost no knowledge, however, on the dynamical mechanism to produce the sentence series. Second, the tools are developed based upon probability theory, which requires the sentence series has an infinite length, at least it should be long enough. But a segment of sentence series has only a limited length (several hundreds). This finite length may induce unreasonable errors or even mistakes to the statistical quantities. Third, a segment of sentence series is generally non-stationary.

More than ten years ago, a concept called diffusion entropy analysis (DEA) [31–33] was proposed to overcome the dynamical mechanism dependent problem, in which instead of the variance one calculates from the PDF of displacements the Shannon entropy at different scales. It is proved that the entropy has a linear relation with the natural logarithm of the scale *s*, the slope of which is the scaling exponent *δ*. Detailed works prove its powerful in evaluation of scaling exponents for time series produced by different mechanisms [34–50]. In a very recent paper, Bonachela et al. [51] proposed an estimator of entropy that performs well when the size of a data set is very small (several tens). The key idea is to balance the bias and fluctuation to be sure their errors are all acceptable. Replacing the original definition of entropy in the DEA method with the balanced estimator, we proposed a new method called balanced estimator of diffusion entropy (cBEDE) [52–55]. Calculations with a large amount of theoretical and empirical data show that it can give us a reliable estimation of scaling behavior embedded in a very short time series with a length of ∼ 10^2^. To our knowledge, at present the cBEDE is the sole method that can obtain a reliable scaling behavior embedded in very short time series with several hundreds a length.

Consequently, in the present paper we are interested in the evolution of scaling behaviors embedded in sentence series. A long sentence series is separated into non-overlapping segments with a predefined length. Scaling behavior of every segment is employed as representative of the language’s characteristics within the corresponding duration. Specially, the classical novel entitled *A Story of the Stone* is investigated to monitor the change of language habit in a more than ten years duration. We hope also to find some new clues on the author’s attribution problem. Each segment covers a total of 500 sentences. To obtain reliable scaling behaviors from so short a time series, the cBEDE is the natural and the sole selection of method.

It is found that the total, the part attributed to Xueqin Cao and the other part attributed to E Gao display almost perfect scale invariance in a wide scale interval of ∼ 10^3^ sentences, while their scaling exponents are almost identical. All the segments of sentence series behave scale invariant in considerable wide scales, most of which can reach $\frac{1}{3}$ of the length. In the curve of scaling exponent versus segment identification number, the part attributed to E Gao is a *U*-shaped valley with a wide low bottom, which supports the current opinion of the author’s attribution of this part. The part attributed to Xueqin Cao, however, contains rich patterns, the values of scaling exponents of which are averagely larger.

## Materials and methods

### Data

*A Story of the Stone* is one of the four greatest Chinese classical vernacular novels [19]. By means of a huge cast of characters and psychological scope, it illustrates precisely the details of life and social structure in the 18th century China. It was completed in a long duration covering approximately 10 years from 1749 to 1759. It was published anonymously until the beginning of the 20th century, which left a famous mystery to the present people, i.e., the author’s attribution. The current opinion is that the part from the 1st to the 80th chapter was written by Xueqin Cao, and the remaining totally 40 chapters were written by E Gao, though there are still some debates.

The text can be downloaded freely (see the Data Availability Statement). From the original text we identify the positions of full stops (“.”), question marks (“?”), exclamation marks (“!”) and suspension points (“……”), as being the stop of a sentence. Increments between the successive positions of the identified stops form the sentence series, an element of which is the number of characters contained in the corresponding sentence. The sentence series for the whole text contains a total of 34,759 elements, in which the part attributed to Xueqin Cao (called X-part) has a length of 23,504 elements and the other part attributed to E Gao (called E-part) has a length of 11,255 elements.

### Correlation dependent balanced estimation of diffusion entropy analysis

If a sentence series behaves scale invariant, as defined in Eq (4), a straightforward computation leads to a linear relation of Shannon entropy versus the natural logarithm of the scale *s* [33],

$$E\left(s\right)\equiv -\int_{-\infty }^{+\infty }p\left(x,s\right)lnp\left(x,s\right)dx∼A+\delta lns.$$ (5)

The slope of this relation equals to *δ*, which is independent with the specific dynamical mechanism the language obeys. It is called diffusion entropy analysis (DEA) [31–33], because the probability, *p*(*x*, *s*), is obtained from a diffusion process.

The relation in Eq (5) stands only for stationary sentence series, which is generally not the case in reality. Herein, the central moving average method [56–59] is used to extract the trend in the displacement series, *x*(*s*) = {*x*_1_(*s*), *x*_2_(*s*), ⋯, *x*_*N*−*s*+1_(*s*)}. Let a window with a size of *s* slide along this series. The trend for the element at the center of the window, i.e., the $[\frac{s+1}{2}]\text{th}$ displacement, is regarded to be the average of all the elements covered by the window, where [⋅] is the integer part of a real number. Subtracting the trend series from the displacement series produces the de-trended displacement series with a length of *N* − 2*s* + 1 elements.

From the de-trended displacement series, let us estimate the PDF. Selecting a certain fraction of the standard deviation of the sentence series *ξ*, $\frac{1}{\epsilon }std\left(\xi \right)$, as being the size of a bin, one can separate the distribution interval of the de-trended displacement series into a total of *R*(*s*) bins. Reckoning the number of the elements in the de-trended series that occur in every bin, denoted with *n*(*k*, *s*), *k* = 1, 2, ⋯, *R*(*s*), the occurring probability in the bins can be approximated with,

$$p\left(k,s\right)\approx \frac{n(k,s)}{N-2s+1},k=1,2,\cdots ,R\left(s\right).$$ (6)

The Shannon entropy is then estimated simply using,

$${\hat{E}}_{\text{sim}}\left(s\right)=-\sum_{k=1}^{R(s)}\frac{n(k,s)}{N-2s+1}ln\;\left[\frac{n(k,s)}{N-2s+1}\right].$$ (7)

If ${\hat{E}}_{\text{sim}}$ obeys the relation in Eq (5), the sentence series is scale invariant.

Though the estimation of *p*(*k*, *s*), *k* = 1, 2, ⋯, *R*(*s*) is unbiased, the estimation of diffusion entropy ${\hat{E}}_{\text{sim}}$ is biased due to its being a nonlinear function of the estimated probabilities. A rough estimation shows us that it will under-estimate the entropy, the bias of which reads $-\frac{R(s)-1}{2(N-2s+1)}+O\left[R\left(s\right)\right]$ [60]. With the increase of *s*, the total number of elements (*N* − 2*s* + 1) decreases, while the number of bins *R*(*s*) increases according to *s*^*δ*^. Because a segment of sentence series is generally very short (several hundreds a length), increase of *s* will lead to unreasonable large bias to the simple estimation of entropy. Hence, an estimator with an acceptable high-performance is required.

Let us denote the perfect occurring probabilities in the bins with *p*(*k*, *s*), *k* = 1, 2, ⋯, *R*(*s*). For a total of *N* − 2*s* + 1 displacements (de-trended), the expected numbers in the bins are (*N* − 2*s* + 1)*p*(*k*, *s*), *k* = 1, 2, ⋯, *R*(*s*). The occurring numbers of *n*(*k*, *s*), *k* = 1, 2, ⋯, *R*(*s*) are a realization of the perfect PDF. The deviations of the realizations from the expected occurring numbers come from uncorrelated statistical noises. A simple constraint is that the total number occurring in all the bins keeps constant. Hence, a correction of the simple estimation in Eq (7) should make the bias and the statistical fluctuation reach simultaneously minima. By a procedure of minimizing the statistical average of the summation of bias and standard deviation of the estimated diffusion entropy, a tedious computation leads to a new estimator,

$${\hat{E}}_{\text{cBEDE}}\left(s\right)=\frac{1}{N-2s+1+R(s)}\sum_{k=1}^{R(s)}\left[n\left(k,s\right)+1\right]\sum_{j=n(k,s)+2}^{N-2s+1+M(s)}\frac{1}{j},$$ (8)

called correlation dependent balanced estimation of diffusion entropy (cBEDE) [55]. Detailed calculations show that it can give us a reliable estimation of scaling exponent from a very short time series with several hundreds a length (e.g., for a trajectory of the fractional Brownian motion, if the length is 300, the bias is less than 0.03 and the standard deviation is about 0.05). Accordingly, it is employed in this work to evaluate scaling behaviors embedded in the segments of sentence series.

To determine the size of a bin, we select different values of *ϵ* (such as 2, 3 and 4) and plot the curve of ${\hat{E}}_{\text{cBEDE}}$ versus *lns* corresponding to every *ϵ*. One will find a special interval of *ϵ*, in which the curves degenerate almost to an identical curve under an operation of vertical shift. The estimated scaling exponent is *ϵ*-independent and should be a correct estimation. In calculations *ϵ* is selected to be 2.

In the scaling behavior, the scale ranges of, for instance, *e*^0^-*e*^1^, *e*^1^-*e*^2^, *e*^2^-*e*^3^ and *e*^3^-*e*^4^ should have the same contributions in estimating the Hurst exponent, though the covered range increases exponentially [18]. In calculations, the window sizes are selected to be *s* = [1.2^*m*^], *m* = 1, 2, ⋯, and $s\leq \frac{N}{3}$, where [·] is the integer part of a real number. The points in the curve of ${\hat{E}}_{\text{cBEDE}}$ versus *lns* distribute at almost identical intervals. All the scales contain almost the same number of points and consequently have identical contributions in the regression procedure of the least square method.

If a sentence series behaves scale invariant, the distribution region of displacement will extends according to ∼ *s*^*δ*^. Hence, if we select the bin size in the procedure of estimating the PDF to be a certain fraction of the standard deviation of the de-trended displacements at time *s* (i.e, the size will increase with *s*), the diffusion entropy should keep constant. Slight deviations from this constant comes from the algorithm and noises, which have been corrected in the final results of ${\hat{E}}_{\text{cBEDE}}$ [55].

### Power spectrum

The scaling behavior of the sentence series in Eq (1) can also be displayed simply with its power spectrum [61], *S*(*f*). For simplicity, we denote the series *ξ* = {*ξ*_1_, *ξ*_2_, ⋯, *ξ*_*N*_} with *ξ* = {*ξ*_0_, *ξ*_2_, ⋯, *ξ*_*N*−1_}, the discrete Fourier transform (DFT) of which reads,

$$l\left(f\right)=\sum_{n=0}^{N-1}\xi_{n}e^{-inf}\equiv \sum_{n=0}^{N-1}\xi_{n}e^{-in\frac{2\pi J}{N}},J=1,2,\cdots ,N-1,$$ (9)

where $f=\frac{2\pi \cdot 0}{N},\frac{2\pi \cdot 1}{N},\frac{2\pi \cdot 2}{N},\cdots ,\frac{2\pi \cdot (N-1)}{N}$ are the frequencies. The power spectrum writes,

$$S\left(f\right)\equiv {\left|l\left(f\right)\right|}^{2}.$$ (10)

An existence of scale invariance implies a power-law, *S*(*f*) ∼ *f*^−*β*^. The relation between *β* and *δ* reads,

β=2δ-1. (11)

The power spectrum is sensitive to noise and trends in the sentence series. The length of the series should also be long enough to obtain a reliable result. In the present paper we calculate also the power spectra for the total, X-part and E-part series, to show the clues of scaling behaviors. The Welch’s method [61] is used to reduce the impacts of noises caused by imperfect and finite data.

## Results

**Fig 1:**
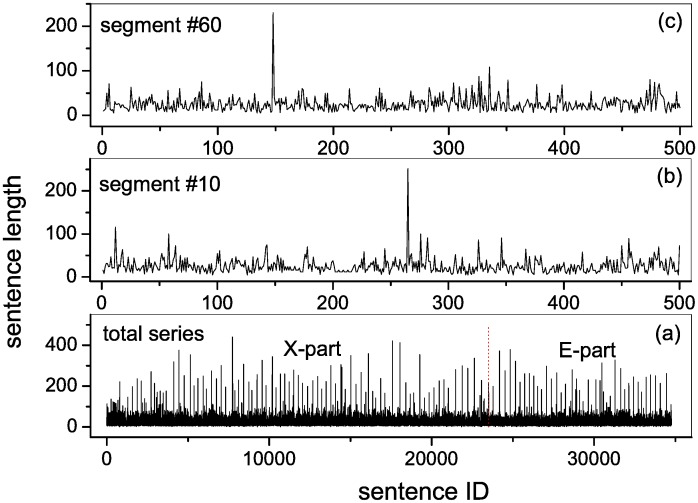
Sentence series. (a) The total sentence series. Every element is the sentence length, i.e, the number of characters covered by the corresponding sentence. The two parts written by Xueqin Cao (X-part) and E Gao (E-part) are separated by a vertical red dotted line. (b) The segment of sentence series numbered *seg*10. (c) The segment of sentence series numbered *seg*60. The total sentence series is separated into a total of 69 non-overlapping segments with a length of 500 sentences each, numbered with *seg*01, *seg*02, ⋯, *seg*69 respectively.

From the original text of *A Story of the Stone*, we identify the positions of specific punctuation marks indicating the end of a sentence. The number of characters (including punctuation marks) between every pair of successive positions is the length of the corresponding sentence. Recording sequentially the length of every sentence in the text produces the sentence series. Fig 1(a) presents the total sentence series, in which the two parts written by Xueqin Cao (X-part) and E Gao (E-part) are separated by a vertical red dotted line. To monitor the evolutionary behavior, we separate the sentence series into a total of 69 successive non-overlapping segments, denoted with *seg*01, *seg*02, ⋯, *seg*69, respectively. Each segment has a length of 500 sentences. We illustrate the segments numbered *seg*10 in Fig 1(b) and *seg*60 in Fig 1(c), as typical examples.

**Fig 2:**
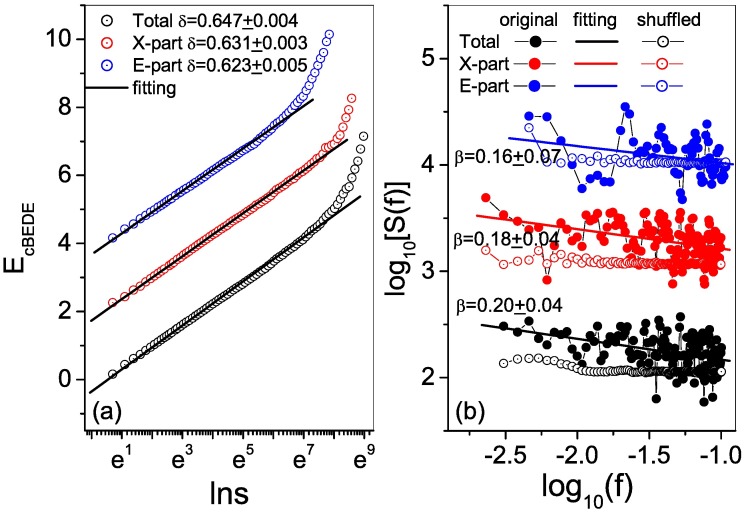
Scaling behaviors of the total, X-part and E-part series. (a) Correlation dependent balanced estimation of diffusion entropies. The scaling exponents for the three series are undistinguishable. (b) Power spectra for the three series. The estimated values of *β* are very close with that of 2*δ* − 1, respectively. The curves are vertically shifted for visual convenience.

Fig 2(a) provides the curves of ${\hat{E}}_{\text{cBEDE}}$ versus *lns* for the total, X-part and E-part series, whose scale ranges in which the scale invariance exists are [*e*^0^, *e*^7^], [*e*^0^, *e*^8^] and [*e*^0^, *e*^7^], covering about $\frac{1}{31}$, $\frac{1}{8}$ and $\frac{1}{10}$ of the corresponding series lengths, respectively. The scaling exponents for the total, X-part and E-part series are 0.65, 0.63 and 0.62, which are almost identical. Hence, the global behaviors can not give us a distinguishable difference between the three series. We calculate also the power spectra of the three series (see Fig 2(b)). One can find that, roughly speaking, they all behave power-law, though the the points scattered in wide regions. The estimated values of *β* are for the total, X-part and E-part are *β* = 0.20 ± 0.04, 0.18 ± 0.04 and 0.16 ± 0.07, which are very close with the corresponding values of 2*δ* − 1 = 0.3, 0.26 and 0.24 respectively. The shuffled series have a scaling exponent of *β* ∼ 0.

**Fig 3:**
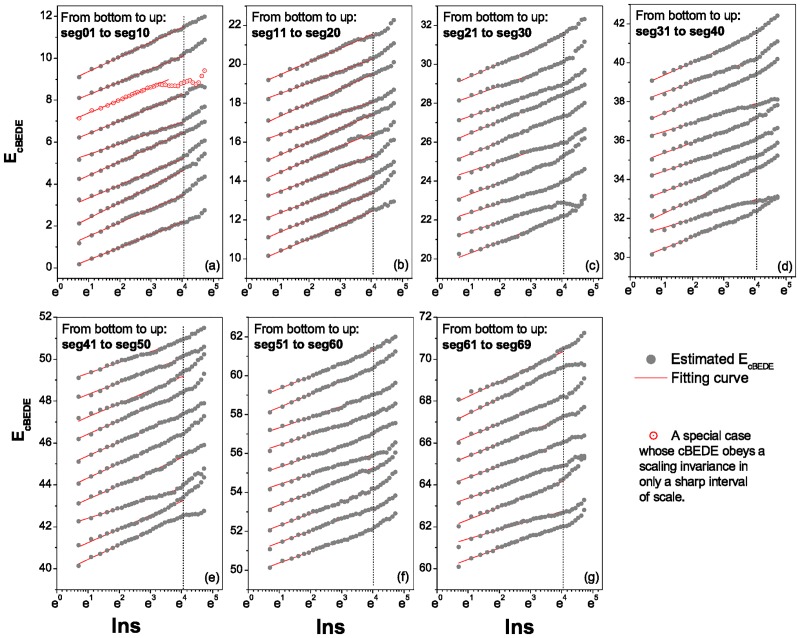
Correlation dependent balanced estimation of diffusion entropies for all the segments. (a)-(g) the segments from *seg*01 to *seg*69. Except few segments such as the segment numbered *seg*08 (red open circles in (a)), all the curves show almost a perfect linear relation of ${\hat{E}}_{\text{cBEDE}}$ versus *lns* in a wide range of scale. For some segments the scale range can reach $\frac{1}{3}$ of the length. The values of slope are all calculated in the scale range of [*e*^0^, *e*^4.1^], covering about $\frac{1}{10}$ of the length. As for the segment numbered *seg*08, the scale range is selected to be [*e*^0^, *e*^3.3^], covering about $\frac{1}{20}$ of the length. The curves are vertically shifted for visual convenience.

Fig 3 displays the curves of ${\hat{E}}_{\text{cBEDE}}$ versus *lns* for all the segments (grey solid circles). One can find that except few segments such as the *seg*08 the red open circles in Fig 3(a) all the curves obey the scaling invariant relation in Eq (5) in considerable wide intervals, some of which can reach $\frac{1}{3}$ of the length. Herein, we evaluate the scaling exponents (slopes of the curves) in the scale interval of [*e*^0^, *e*^4.1^] covering about $\frac{1}{10}$ of the series length. Here we do not show the power spectra of the segments, though we have noticed some clues for scale invariant behaviors. One can not expect a reliable power spectrum for a series containing only 500 records.

**Fig 4:**
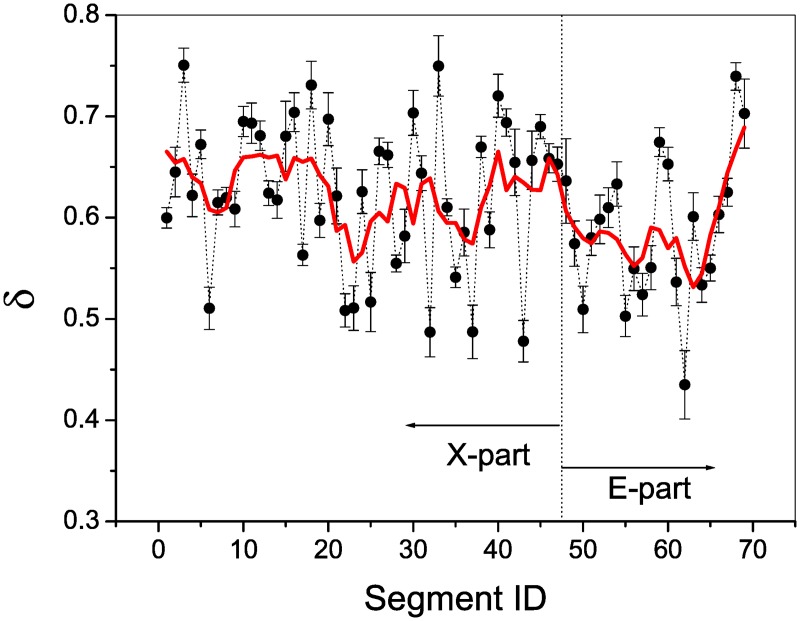
Evolution of scaling invariance. With the increase of the segment number, the scaling exponent changes abruptly in a wide interval of [0.43, 0.75]. Let a window covering 5 scaling exponents slide along the curve, and replace each value at the center of the window with the average of the covered scaling exponents. This smoothing procedure results in the smoothing curve (the red curve). When the segment number becomes larger than 47 (from then on the segments belong to the E-part), the smoothing curve has a *U*-shape with a wide bottom. In the E-part, the curve contains rich patterns, and the scaling exponents are comparatively larger.

As shown in Fig 4 the scaling exponents for all the segments, one can find that with the increase of the segment number the scaling exponent changes abruptly in a wide interval of [0.43, 0.75]. To obtain the global behavior, we conduct a smooth procedure, namely, let a window covering five scaling exponents slide along the curve and replace the scaling exponent at the center with the average of the covered scaling exponents. The resulting curve is regarded as the smoothing curve (see the red curve). Interestingly, when the segment number becomes larger than 47, i.e., from then on the segments belong to the E-part, the smoothing curve decreases rapidly to a small value of about 0.57 at the 51th segment, then oscillates around the small value up to the 65th segment, and finally increases in a speedy way to about 0.70 at the last segment. By this way the smoothing curve shows a significant wide valley in the E-part. As for the X-part, though there exist rich structural patterns, averagely the scaling exponents are comparatively larger.

To be sure the detected scaling behaviors are dependent with sentence order, we calculate also the scaling exponents for the shuffled sentence series and the shuffled segments. The scaling exponents are almost identical (≈ 0.5) with confidence intervals within [−0.05, +0.05] (not shown).

## Summary and conclusion

The novel entitled *A Story of the Stone* provides us details of life and structure of social society in the 18th century of China, and attracts subsequently attentions from diverse research fields. In the present paper, we focus our attention on the scaling behaviors embedded in the sentence series from this novel, from which we hope to find how the description is structured and constructed from microscopic to macroscopic level, i.e., from single sentences, to paragraphs, chapters and the final whole text. This novel was completed in a long duration lasting about ten years, in which the author’s language habit might change significantly. It had been published anonymously up to the beginning of the 20th century, which left a famous mystery of author’s attribution. Hence, we are interested specially on how the scaling behavior evolves with time, and on if the scaling behavior can provides us much more clues on debates of the author’s attribution.

To obtain the evolutionary behavior we separate the whole text into many non-overlapping segments, and take the scaling behavior embedded in every segment as the representative of the behavior in the corresponding time interval. In literature, there are several standard tools to evaluate the scaling behaviors embedded in time series, such as the WTMM, the DFA, and the DEA. These tools are designed based upon probability theory, which requires the time series having an infinite length (at least the length is long enough). However, in our work we separate the sentence series into a total of 69 segments with a limited length of 500 sentences each. Very recently, we developed a new concept called cBEDE to obtain reliable scaling behaviors embedded in very short time series. Calculations on a large amount of fractional Brownian motions and empirical records from stock markets and physiological experiments prove its high performance. For instance, for time series with a length of 300 the bias is in the interval of [−0.03, +0.03] and the confidence interval is [−0.05, +0.05]. Accordingly, the cBEDE is employed to monitor the evolution of scaling behavior.

A current opinion is that the part from the 1th to the 80th chapter was written by Xueqin Cao, and the other part from the 81th to the 120th chapter by E Gao, herein denoted with X-part E-part respectively. The total, X-part, and E-part series behave scale invariant in a considerable wide interval of scale (up to more than 10^3^ sentences), but the scaling exponents are undistinguishable in value. Hence, the ideas are structured with an identical rule up to a scale of ∼ 10^3^ sentences covering averagely three to four chapters. However, the scaling invariance can not give us any clue on the author’s attribution.

All the segments of sentence series display almost perfect scale invariance, the scale ranges of most of which can reach $\frac{1}{3}$ of the length. The values of scaling exponent distribute in a wide interval of [0.43, 0.75. From the curve of scaling exponent versus the segment number, one can find that the E-part has a *U*-shape with a wide low bottom. This finding gives a new clue to attribute the E-part to E Gao. However, though averagely the curve for the X-part has comparatively larger values of scaling exponent, it has a complicated shape with rich patterns.

Summarily, the scale invariance exists up to a scale of 10^3^ sentences (about three chapters). The scaling behaviors for the X-part and the E-part are undistinguishable from that for the whole novel. However, the scaling behaviors for segments of sentence series display rich structures and significant difference for the X-part and the E-part. Hence, structures at different scales from sentences to chapters can tell us valuable information.

Recent years have witnessed a significant progress in detecting structural patterns of time series. For instance, the unbiased estimator of probability moments [62] can evaluate multi-fractals in very short time series (with a length of ∼ 10^2^). By mapping a time series to a network one can extract the structural patterns at different scales [63–80]. By using a graph-let as the representative of a local state, we can monitor the evolutionary behavior of a complex system [81, 82]. We hope our work stimulates an incorporation of the methods to enrich the knowledge of and to deepen the understanding on the novel of *A Story of The Stone*.

## References

[1] Jackendoff R. Possible stages in the evolution of the language capacity. Trends Cogn. Sci. 1999 7;3(7):272–279. 10.1016/S1364-6613(99)01333-9 10377542

[2] Hauser MD, Chomsky N, Fitch WT. The faculty of language: what is it, who has it, and how did it evolve? Science 2002 11;298:1569–1579. 10.1126/science.298.5598.1569 12446899

[3] Peter F, Alfred T. Toward a phylogenetic chronology of ancient Gaulish, Celtic, and Indo-European. Proc. Natl. Acad. Sci. USA 2003 7;100:9079–9084. 10.1073/pnas.1331158100 12837934 PMC166441

[4] Russell DG, Quentin DA. Language-tree divergence times support the Anatolian theory of Indo-European origin. Nature 2003 11;426:435–439. 10.1038/nature02029 14647380

[5] Zipf GK. Human Behavior and the Principle of Least Effort. Addison-Wesley, Cambridge; 1949.

[6] Laherrere J, Sornette D. Stretched exponential distributions in nature and economy: fat tails with characteristic scales. Eur. Phys. J. B 1998 4;2(4):525–539. 10.1007/s100510050276

[7] Altmann EG, Pierrehumbert JB, Motter AE. Beyond word frequency: bursts, lulls, and scaling in the temporal distributions of words. Plos ONE 2009 11;4(11):e7678 10.1371/journal.pone.0007678 19907645 PMC2770836

[8] Montemurro MA, Pury PA. Long-range fractal correlations in literary corpora. Fractals 2002 12;10:451–461. 10.1142/S0218348X02001257

[9] Altmann EG, Cristadoro G, Esposti MD. On the origin of long-range correlations in texts. Proc. Natl. Acad. Sci. 2012 7;109:11582–11587. 10.1073/pnas.1117723109 22753514 PMC3406867

[10] Ausloos M. Generalized hurst exponent and multifractal function of original and translated texts mapped into frequency and length time series. Phys. Rev. E 2012 9;86:031108 10.1103/PhysRevE.86.031108 23030867

[11] Ausloos M. Measuring complexity with multifractals in texts: Translation effects. Chaos, Solit. Fract. 2012 11;45(11):1349–1357.

[12] Cancho RF-i, Sole RV. The small world of human language. Proc. Roy. Soc. Lond. B 2001 11;268:2261–2265. 10.1098/rspb.2001.1800 PMC1088874 11674874

[13] Cong J, Liu H. Approaching human language with complex networks. Phys. Life Rev. 2014 12;11:598618 And references there-in. 10.1016/j.plrev.2014.04.004 24794524

[14] Kulig A, Drozdz S, Kwapien J, Oswiecimka P. Modeling the average shortest-path length in growth of word-adjacency networks. Phys. Rev. E 2015 3;91:032810 10.1103/PhysRevE.91.032810 25871160

[15] Kulig A, Kwapien J, Stanisz T, Drozdz S. In narrative texts punctuation marks obey the same statistics as words. Information Sciences 2017 1;375:98–113. 10.1016/j.ins.2016.09.051

[16] Mandelbrot BB. The Fractal Geometry of Nature. Freeman, San Francisco; 1982.

[17] Drozdz S, Oswiecimka P, Kulig A, Kwapien J, Bazarnik K, Grabska-Gradzinska I, et al Quantifying origin and character of long-range correlations in narrative texts. Information Sciences 2016 2;331:32–44. 10.1016/j.ins.2015.10.023

[18] Yang TG, Gu CG, Yang HJ. Long-Range Correlations in Sentence Series from *A Story of the Stone*. PLoS ONE 2016 9;11(9):e0162423 10.1371/journal.pone.0162423 27648941 PMC5029871

[19] Zhou RC. Between Noble and Humble: Cao Xueqin and the Dream of the Red Chamber, edited by Ronald RG and Mark SF. New York:Peter Lang; 2009.

[20] Muzy JF, Bacry E, Arneodo A. Wavelets and multifractal formalism for singular signals: Application to turbulence data. Phys. Rev. Lett. 1991 12;67:3515–3518. 10.1103/PhysRevLett.67.3515 10044755

[21] Mallat S, Hwang WL. Singularity detection and processing with wavelets. IEEE Trans. Inform. Theor. 1992 3;38(2):617–643. 10.1109/18.119727

[22] Muzy JF, Bacry E, Arneodo A. Multifractal formalism for fractal signals: The structure-function approach versus the wavelet-transform modulus-maxima method. Phys. Rev. E 1993 2;47:875–884. 10.1103/PhysRevE.47.875 9960082

[23] Peng CK, Buldyrev SV, Havlin S, Simons M, Stanley HE, Goldberger AL. Mosaic organization of DNA nucleotides. Phys. Rev. E 1994 2;49:1685–1689. 10.1103/PhysRevE.49.1685 9961383

[24] Buldyrev SV, Goldberger AL, Havlin S, Mantegna RN, Matsa ME, Peng CK, et al Long-range correlation properties of coding and noncoding DNA sequences: GenBank analysis. Phys. Rev. E 1995 5;51:5084–5091. 10.1103/PhysRevE.51.5084 9963221

[25] Hu K, Ivanov PCH, Chen Z, Carpena P, Stanley HE. Effect of trends on detrended fluctuation analysis. Phys. Rev. E 2001 6;64:011114 10.1103/PhysRevE.64.011114 11461232

[26] Chen Z, Ivanov PCH, Hu K, Stanley HE. Effect of nonstationarities on detrended fluctuation analysis. Phys. Rev. E 2002 4;65:041107 10.1103/PhysRevE.65.041107 12005806

[27] Stanley HE, Kantelhardt JW, Zschiegner SA, Koscielny-Bunde E, Havlin S, Bunde A. Multifractal detrended fluctuation analysis of nonstationary time series. Physica A 2002 12;316:87–114. 10.1016/S0378-4371(02)01383-3

[28] Podobnik B, Stanley HE. Detrended Cross-Correlation Analysis: A New Method for Analyzing Two Nonstationary Time Series. Phys. Rev. Lett. 2008 2;100:084102 10.1103/PhysRevLett.100.084102 18352624

[29] Podobnik B, Horvatic D, Petersen AM, Stanley HE. Cross-correlations between volume change and price change. Proc. Natl. Acad. Sci. 2009 12;106:22079–22084. 10.1073/pnas.0911983106 20018772 PMC2799689

[30] Xiao Q, Pan X, Mutua S, Yang Y, Li XL, Yang HJ. Discrete scale-invariance in cross-correlations between time series. Physica A 2015 3;421:161–170. 10.1016/j.physa.2014.11.032

[31] Scafetta N, Hamilton P, Grigolini P. The thermodynamics of social processes: The teen birth phenomenon. Fractals 2001 6;9(2):193–208. 10.1142/S0218348X0100052X

[32] Grigolini P, Palatella L, Raffaelli G. Asymmetric anomalous diffusion: an efficient way to detect memory in time series. Fractals 2001 12;9:439–449. 10.1142/S0218348X01000865

[33] Scafetta N, Grigolini P. Scaling detection in time series: Diffusion entropy analysis. Phys. Rev. E 2002 9;66:036130 10.1103/PhysRevE.66.036130 12366207

[34] Scafetta N, West BJ. Multiscaling Comparative Analysis of Time Series and a Discussion on Earthquake Conversations in California. Phys. Rev. Lett. 2004 4;92:138501 10.1103/PhysRevLett.92.138501 15089646

[35] Scafetta N, West BJ. Solar flare intermittency and the Earth’s temperature anomalies. Phys. Rev. Lett. 2003 6;90:248701 10.1103/PhysRevLett.90.248701 12857233

[36] Scafetta N, Grigolini P, Imholt T, Roberts J, West BJ. Solar turbulence in earth’s global and regional temperature anomalies. Phys. Rev. E 2004 2;69:026303 10.1103/PhysRevE.69.026303 14995555

[37] Acquisti C, Allegrini P, Bogani P, Buiatti M, Catanese E, Fronzoni L, et al In the search for the low-complexity sequences in prokaryotic and eukaryotic genomes: how to derive a coherent picture from global and local entropy measures. Chaos, Solitons, and Fractals 2004 4;20,127–137.

[38] Yang HJ, Zhao FC, Qi LY, Hu BL. Temporal series analysis approach to spectra of complex networks. Phys. Rev. E 2004 6;69:066104 10.1103/PhysRevE.69.066104 15244664

[39] Scafetta N, West BJ. Multiscaling comparative analysis of time series and geophysical phenomena. Complexity 2005 3;10,51–56.

[40] Yang H, Zhao F, Zhang W, Li Z. Diffusion Entropy Approach to Complexity of a Hodgkin-Huxley Neuron. Physica A 2005 3;347:704–710. 10.1016/j.physa.2004.08.017

[41] Perello J, Montero M, Palatella L, Simonsen I, Masoliver J. Entropy of the Nordic electricity market: anomalous scaling, spikes, and mean-reversion. J. Stat. Mech.: Theor. Exper. 2006,11;2006(11):P11011 10.1088/1742-5468/2006/11/P11011

[42] Cai SM, Zhou PL, Yang HJ, Yang CX, Wang BH, Zhou T. Diffusion entropy analysis on the scaling behavior of financial markets. Physica A 2006 7;367:337–344. 10.1016/j.physa.2005.12.004

[43] Zhao FC, Yang HJ, Wang BH. Complexities of human promoter sequences. J. Theor. Bio. 2007 8;247:645–649. 10.1016/j.jtbi.2007.03.035 17482648

[44] Scafetta N, Moon R, West BJ. Fractal Response of Physiological Signals to Stress Conditions, Environmental Changes, and Neurodegenerative Diseases. Complexity 2007 5;12:12–17. 10.1002/cplx.20183

[45] Cai SM, Zhou PL, Yang HJ, Zhou T, Wang BH, Zhao FC. Diffusion entropy analysis on the stride interval fluctuation of human gait. Physica A 2007 3;375:687–692. 10.1016/j.physa.2006.10.027

[46] Tsai CY, Shieh CF. A study of the time distribution of inter-cluster earthquakes in Taiwan Physica A 2008,9;387:5561–5566. 10.1016/j.physa.2008.05.023

[47] Scafetta N, Marchi D, West BJ. Understanding the complexity of human gait dynamics. Chaos 2009 6;19:026108 10.1063/1.3143035 19566268

[48] Scafetta N, West BJ. Comment on ‘Testing hypotheses about Sun-climate complexity linking’. Phys. Rev. Lett. 2010 11;105:218801 10.1103/PhysRevLett.105.219801 21231365

[49] Scafetta N. Diffusion Entropy Analysis of Time Series: Theory, concepts, applications and computer codes for studying fractal noises and Levy walk signals. VDM Verlag Dr. Mller; 7 2010.

[50] Gao ZK, Yang YX, Zhai LS, Ding MS, Jin ND. Characterizing slug to churn flow transition by using multivariate pseudo Wigner distribution and multivariate multiscale entropy. Chem. Engi. J. 2016 5;291:74–81. 10.1016/j.cej.2016.01.039

[51] Bonachela JA, Hinrichsen H, Munoz MA. Entropy estimates of small data sets. J. Phys. A: Math. Theor. 2008 4;41:202001 10.1088/1751-8113/41/20/202001

[52] Qi JC, Yang HJ. Hurst exponents for short time series. Phys. Rev. E 2011 12;84:066114 10.1103/PhysRevE.84.066114 22304162

[53] Zhang WQ, Qiu L, Xiao Q, Yang HJ, Zhang QJ, Wang JY. Evaluation of scale invariance in physiological signals by means of balanced estimation of diffusion entropy. Phys. Rev. E 2012 11;86:056107 10.1103/PhysRevE.86.056107 23214843

[54] Pan X, Hou L, Stephen M, Yang HJ. Long-term memories in online users’ selection activities. Phys. Lett. A 2014 7;378(35):2591–2596. 10.1016/j.physleta.2014.07.012

[55] Pan X, Hou L, Stephen M, Yang HJ, Zhu CP. Evaluation of scaling invariance embedded in short time series. Plos ONE 2014 12;9(12):e116128 10.1371/journal.pone.0116128 25549356 PMC4280174

[56] Alessio E, Carbone A, Castelli G, Frappietro V. Second-order moving average and scaling of stochastic time series. Eur. Phys. J. B 2002 5;27:197–200.

[57] Xu L, Ivanov PCh, Hu K, Carbone A, Stanley HE. Quantifying signals with power-law correlations: A comparative study of detrended fluctuation analysis and detrended moving average techniques. Phys. Rev. E 2005 5;71:051101 10.1103/PhysRevE.71.051101 16089515

[58] Jiang ZQ, Zhou WX. Multifractal detrending moving-avarage cross-correlation analysis. Phys. Rev. E 2011 7;84:016106 10.1103/PhysRevE.84.016106 21867256

[59] Gao ZK, Fang PC, Ding MS, Jin ND. Multivariate weighted complex network analysis for characterizing nonlinear dynamic behavior in two-phase flow. Exp. Therm. & Flu. Sci. 2015 1;60:157–164. 10.1016/j.expthermflusci.2014.09.008

[60] Roulston MS. Estimating the errors on measured entropy and mutual information. Physica D 1999 1;125:285–294. 10.1016/S0167-2789(98)00269-3

[61] Welch PD. The use of Fast Fourier Transform for the estimation of power spectra: A method based on time averaging over short, modified periodograms. Trans. Audio Electroacoust. 1967 6;15(2):70–73. 10.1109/TAU.1967.1161901

[62] Qiu L, Yang TG, Yin YH, Gu CG, Yang HJ. Multifractals embedded in short time series: An unbiased estimation of probability moment. Phys. Rev. E 2016 12; 94, 062201 10.1103/PhysRevE.94.062201 28085321

[63] Zhang J, Small M. Complex network from pseudoperiodic time series: topology versus dynamics. Phys. Rev. Lett. 2006 6;96:238701 10.1103/PhysRevLett.96.238701 16803415

[64] Yang Y, Yang H. Complex network based time series analysis. Physica A 2008 2; 387:1381–1386. 10.1016/j.physa.2007.10.055

[65] Lacasa L, Luque B, Ballesteros F, Luque J, Nuno JC. From time series to complex networks: The visibility graph. Proc. Natl. Acad. Sci. (USA) 2008 4;105(13):4972–4975. 10.1073/pnas.0709247105 18362361 PMC2278201

[66] Xu X, Zhang J, Small M. Superfamily phenomena and motifs of networks induced from time series. Proc. Natl. Acad. Sci. (USA) 2008 12;105(50):19601–19605. 10.1073/pnas.0806082105 19064916 PMC2604928

[67] Donner RV, Zou Y, Donges JF, Marwan N, Kurths J. Recurrence networks—a novel paradigm for nonlinear time series analysis. New J. Phys. 2010 3;12(3):033025 10.1088/1367-2630/12/3/033025

[68] Gao ZK, Jin ND. A directed weighted complex network for characterizing chaotic dynamics from time series. Nonlinear Analysis: Real World Appllications 2012 4;13:947–952. 10.1016/j.nonrwa.2011.08.029

[69] Gao YC, Wei ZW, Wang BH. Dynamic evolution of financial network and its relation to economic crisis Int. J. Mod. Phys. C 2013,2;24(2):135005 10.1142/S0129183113500058

[70] Gao ZK, Zhang XW, Jin ND, Marwan N, Kurths J. Multivariate recurrence network analysis for characterizing horizontal oil-water two-phase flow. Phys. Rev. E 2013 12;88:032910 10.1103/PhysRevE.88.032910 24125328

[71] Xiao Q, Pan X, Li XL, Stephen M, Yang H, Jiang Y, et al Row-column visibility graph approach to two-dimensional landscapes. Chin. Phys. B 2014 7;23(7):078904 10.1088/1674-1056/23/7/078904

[72] Gao ZK, Yang YX, Fang PC, Jin ND, Xia CY, Hu LD. Multi-frequency complex network from time series for uncovering oil-water flow structure. Sci. Rept. 2015 2;5:8222 10.1038/srep08222 PMC4316157 25649900

[73] Xu WJ, Zhong LX, Huang P, Qiu T, Zhong CY. Evolutionary dynamics in opinion formation model with coupling of social communities. Adv. Compl. Syst. 2015 2;18(01n02):1550003 10.1142/S0219525915500034

[74] Gao ZK, Yang YX, Fang PC, Zou Y, Xia CY, Du M. Multiscale complex network for analyzing experimental multivariate time series. Europhys. Lett. 2015 2; 109(3): 30005 10.1209/0295-5075/109/30005

[75] Chen G, Qiu T, Jiang XF, Zhong LX, Wu XR. How trading volume responds to return in financial dynamics? Physica A 2015 4;424:73–81. 10.1016/j.physa.2015.01.001

[76] Zhao ZD, Cai SM, Lu Y. Non-markovian character in human mobility: Online and offline. Chaos 2015 6;25:063106 10.1063/1.4922302 26117100

[77] Gao ZK, Yang YX, Zhai LS, Jin ND, Chen GR. A Four-Sector Conductance Method for Measuring and Characterizing Low-Velocity Oil–Water Two-Phase Flows. IEEE Trans. Instrum. Meas. 2016 7; 65(7): 1690–1697. 10.1109/TIM.2016.2540862

[78] Gao ZK, Cai Q, Yang YX, Dang WD, Zhang SS. Multiscale limited penetrable horizontal visibility graph for analyzing nonlinear time series. Sci. Rept. 2016 10; 6:35622 10.1038/srep35622 PMC5069474 27759088

[79] Zhao ZD, Gao YC, Cai SM, Zhou T. Dynamic patterns of academic forum activities. Physica A 2016 11;461:117–124. 10.1016/j.physa.2016.05.033

[80] Gao ZK, Cai Q, Yang YX, Dong N, Zhang SS. Visibility Graph from Adaptive Optimal Kernel Time-Frequency Representation for Classification of Epileptiform EEG. Int. J. Neur. Syst. 2017 6; 27(4):1750005. 10.1142/S0129065717500058 27832712

[81] Mutua S, Gu CG, Yang HJ. Visibility Graph Based Time Series Analysis. Plos ONE 2015 11;10(11):e0143015 10.1371/journal.pone.0143015 26571115 PMC4646626

[82] Mutua S, Gu CG, Yang HJ. Visibility Graphlet Approach to Chaotic Time Series. Chaos 2016 5;26(5):053107 10.1063/1.4951681 27249947

